# Does Machine Understanding Require Consciousness?

**DOI:** 10.3389/fnsys.2022.788486

**Published:** 2022-05-18

**Authors:** Robert Pepperell

**Affiliations:** Fovolab, Cardiff Metropolitan University, Cardiff, United Kingdom

**Keywords:** machine learning, consciousness, naturalism, understanding, brain modelling

## Abstract

This article addresses the question of whether machine understanding requires consciousness. Some researchers in the field of machine understanding have argued that it is not necessary for computers to be conscious as long as they can match or exceed human performance in certain tasks. But despite the remarkable recent success of machine learning systems in areas such as natural language processing and image classification, important questions remain about their limited performance and about whether their cognitive abilities entail genuine understanding or are the product of spurious correlations. Here I draw a distinction between natural, artificial, and machine understanding. I analyse some concrete examples of natural understanding and show that although it shares properties with the artificial understanding implemented in current machine learning systems it also has some essential differences, the main one being that natural understanding in humans entails consciousness. Moreover, evidence from psychology and neurobiology suggests that it is this capacity for consciousness that, in part at least, explains for the superior performance of humans in some cognitive tasks and may also account for the authenticity of semantic processing that seems to be the hallmark of natural understanding. I propose a hypothesis that might help to explain why consciousness is important to understanding. In closing, I suggest that progress toward implementing human-like understanding in machines—machine understanding—may benefit from a naturalistic approach in which natural processes are modelled as closely as possible in mechanical substrates.

## Introduction

The human capacity for understanding is a complex phenomenon that can involve many cognitive processes such as learning, insight, reward, memory, recognition, and perception. To implement this phenomenon mechanically—that is, to create machines that understand in the same way that humans do—presents an extremely daunting challenge.

Significant progress has been made toward this goal in the field of machine learning. We now have systems that perform very well, and sometimes better than humans, in language processing tasks (Devlin et al., 2019; He et al., 2021), image classification tasks (Zelinsky, 2013; Yang et al., 2019), and in playing complex games (Silver et al., 2016). Even though these systems are very effective in some situations, questions remain about how robust and generalisable they are (Shankar et al., 2020) and to what extent they are truly capable of human-like understanding or whether they are just computational manifestations of the Clever Hans spurious correlation effect (Lapuschkin et al., 2019). In the early twentieth century, a horse of that name was touted as being able to solve arithmetic problems but was later found to be responding to involuntary cues in the body language of its trainer (Pfungst, 1911). This concern is related to the long-standing problem of authenticity raised by John Searle’s Chinese Room argument about whether artificially intelligent machines have semantic understanding of the data they are processing or whether they are “blindly” following syntactic rules (Searle, 1984).

This article addresses the question of what constitutes understanding in humans and how it compares to the kind of understanding that is currently being implemented in digital computers. Partially following Les and Les (2017), I draw a distinction between “natural,” “artificial,” and “machine” understanding, as set out in Table 1. Natural understanding is the kind that humans are capable of; it is instantiated in the physical substrate of our nervous systems, in particular in our brains, and is regarded as “authentic.” I take it that this is the kind of understanding that we ultimately aim to implement in machines. Artificial understanding is a kind of understanding that is currently implemented in highly trained digital computers and is exemplified by natural language processors like BERT (Devlin et al., 2019) and image classifiers like AlexNet (Krizhevsky et al., 2017). For the reasons just given, this kind of understanding does not perform as well, and nor is it regarded as authentic as, natural understanding.

**TABLE 1:** Definitions of the three kinds of understanding referred to in this article.

Definitions of kinds of understanding	
Natural understanding	The human-like capacity for understanding that is instantiated in our neurobiology, in particular in our brains
Artificial understanding	The capacity for understanding that is implemented in machine learning algorithms as instantiated in digital computers
Machine understanding	The human-like capacity for natural understanding implemented in a non-human mechanical substrate

I will analyse examples of natural and artificial understanding to describe some of their key properties and then compare these properties in light of the challenge of producing machine understanding, defined here as natural understanding implemented in a mechanical substrate^1^. The analysis suggests that natural understanding is distinguished from artificial understanding by its property of consciousness and that machine understanding systems may require this property if they are to overcome the limitations of current artificial understanding systems. This leads to the formulation of a hypothesis about why the capacity for consciousness is advantageous to natural understanding.

With some exceptions (e.g., Yufik, 2013; Hildt, 2019) recent theorists have argued that it is not a requirement that computer-based systems are capable of consciousness or genuine semantic appreciation in order to understand (e.g., Anderson, 2017; Les and Les, 2017; Thórisson and Kremelberg, 2017; Dietterich, 2019). The primary goal of these theorists is to design machines that perform well in problem solving, object detection, recognition, and language processing tasks (Zelinsky, 2013; Yang et al., 2019). Indeed, based on the levels of performance in these tasks achieved with recent machine learning systems, which are not claimed to be conscious, there is justification for arguing that consciousness is *not* a necessary requirement for artificial understanding, at least in some cases. But if our goal is to create machine understanding, as defined here, then the requirements may be different. Here I consider in more detail what constitutes natural understanding.

## Natural Understanding

Understanding cannot be easily or precisely defined. It has several subtly different senses in English (Oxford English Dictionary) and interpretations can vary from field to field. But is generally taken to mean the ability to “grasp” or “see” how different parts relate to or depend upon each other (Grimm, 2011). In this section I aim to provide a fuller description of some of the key properties of understanding by reference to two concrete examples. To take first a simple example from the domain of natural language understanding, for each of these sets of three words find the fourth word that they have in common:


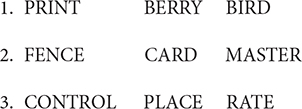


These are examples of the Remote Associates Test commonly used to evaluate cognitive processes such as creative potential, problem solving, divergent thinking, and insight (Mednick, 1968; Bowden and Jung-Beeman, 2003). Consider your train of thought as you find the solution. When you begin the task the three given words seem to form an unrelated sequence. You may feel a mild sense of tension or anxiety as you struggle to find the answer. You probably take each given word in turn and wait for it to trigger other words, jumping between the given words until you alight upon a new word that links all three. Having found the common word, the three given words seem to subtly change their meaning by association with the common word. They acquire a new relationship with each other while retaining their distinct identities. Once you have understood the connection between each set of words you may feel a sudden mild sensation of pleasure or relief^2^.

**FIGURE 1:**
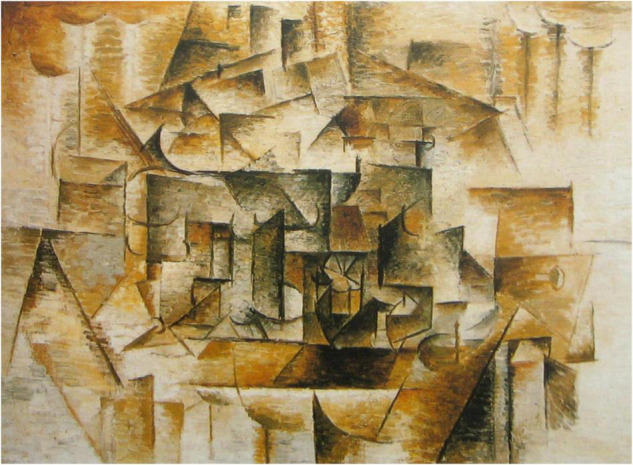
A reproduction of a painting by Pablo Picasso from 1910. ©Succession Picasso/DACS, London 2022.

To take a more involved example from the domain of art interpretation, consider the painting reproduced in Figure 1 that was painted by Pablo Picasso in 1910. It is a typical example of the analytic cubist style, developed by Picasso and Georges Braque in the years before world war I and depicts an arrangement of everyday household objects. If you are unfamiliar with the visual language of cubism it may be very hard—even impossible— to understand what it depicts and it usually takes some training and practice to unpick the objects it contains from the seemingly abstract forms.

**FIGURE 2:**
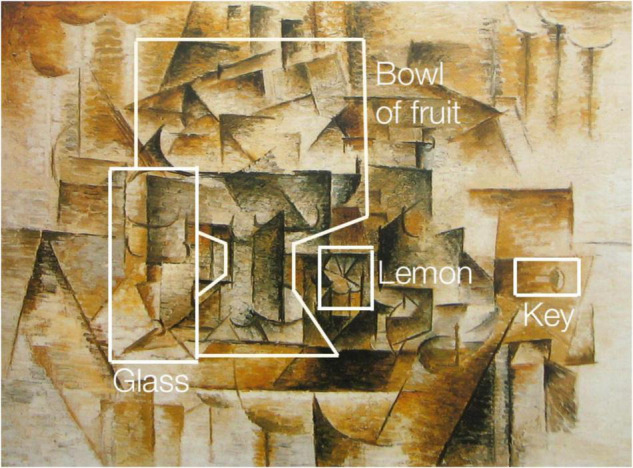
A reproduction of *Still Life with Lemons* by Pablo Picasso from 1910 with outlined and labelled objects. The painting depicts a table containing a number of everyday household items, including glasses, a fruit bowl, a lemon, and a key. The edges and legs of the table can be seen to the left and right of the central grouping of objects.

Now consider the image presented in Figure 2. This shows the same painting, but this time some of the objects have been outlined and labelled. If you study this painting (which is known as “Still Life with Lemons”) and then return to Figure 1 you should now be able to recognise at least some of the items it contains without the guidelines. Given more time and effort you should eventually be able to piece together the entire composition. Arguably, you will then have gained a greater understanding of the meaning of the painting. Perhaps this understanding dawns through a gradual analysis of the relations between objects and their position in space. Or perhaps it appears as a momentary flash of insight—sometimes referred to as an “Aha!” moment—that is accompanied by the feeling of relief or satisfaction associated with a sudden gain of information (Muth and Carbon, 2013; Damiano et al., 2021). Either way, a significant shift has taken place in your perceptual and cognitive faculties such that objects and relationships between objects that were previously absent are now present, despite the fact that you are looking at the same image.

What is going on at the perceptual, cognitive, and phenomenological levels during this acquisition of understanding? Prior to viewing Figure 2 you probably experienced a more or less abstract array of patterns and marks, perhaps attended by a feeling of bewilderment or frustration. Then, using the outline guides provided in Figure 2, you began to separate the boundaries of certain objects from their surroundings until you established their individual identities and how they are spatially positioned in relation to each other and to the scene as a whole. According to the predictive coding theory of object recognition, your brain drew upon high-level cognitive models that influenced the processing of lower-level perceptual input via feedback in order to rapidly anticipate the most probable meaning of what is being perceived (Rao and Ballard, 1999). Once this meaning has been grasped you have created a new network of semantic associations around the image that are grounded in the wider context of your background knowledge and experience (Harnad, 1990).

Understanding, recognition, detection and learning are related but distinct processes. In one sense by studying this image you have learned to detect and classify or label the objects as any machine learning system might be trained to do with sufficient training examples and computer power. But in experiencing the phenomenal Aha! insight that accompanies the understanding you have not just produced a certain statistical output from a certain input; your perceptual, cognitive and phenomenological facilities have undergone a transformation from a state where that meaning is absent to one where it is present. There is evidence from brain imaging and behavioural studies that having undergone this experience with a small number of examples of cubist paintings people are able to recognise more objects more quickly in new examples while undergoing measurable differences in brain activation (Wiesmann et al., 2009)^3^.

**FIGURE 3:**
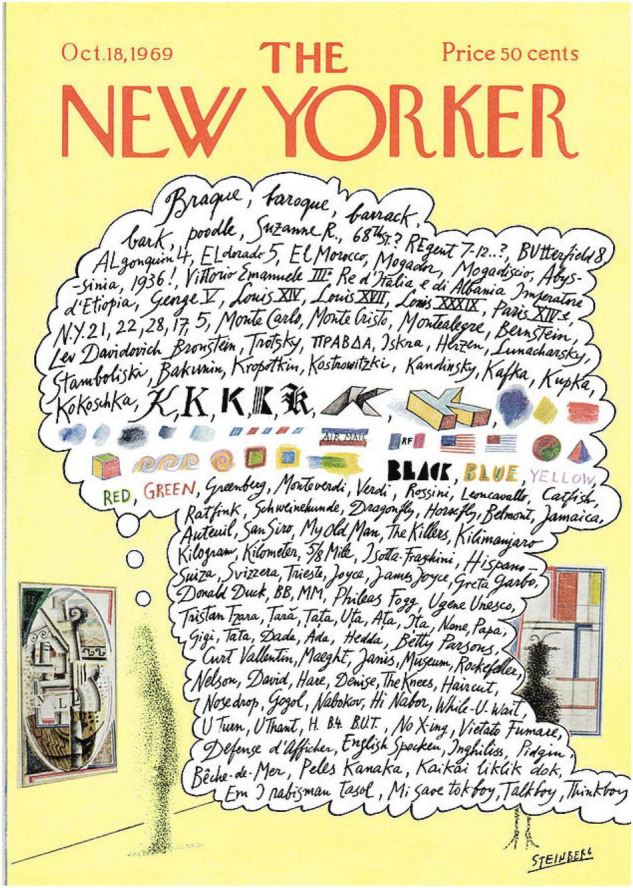
Cover of New Yorker magazine with a cartoon by Saul Steinberg illustrating the diverse train of thought of a person viewing a cubist painting by Georges Braque.

It is also important to stress that acquiring understanding does not merely entail local object detection and recognition but also in holding several distinct concepts in mind at once, along with each of their attendant associations, while forming a global conception of their interrelations and overall significance. These distinct concepts can be highly diverse, as is illustrated in the cartoon by Saul Steinberg that featured on the cover of New Yorker magazine in 1969 showing the train of thought of a person viewing a cubist painting by Georges Braque (Figure 3)^4^. And they are not necessarily logically consistent. So, for example, a certain patch of painting composed of diagonal lines, curves and greyish-brown paint looks very unlike a lemon at the same time as being a lemon. This dichotomy between the material from which an image is constructed (paint, ink, pixels, etc.) and the objects that the material represents is a fundamental feature of all pictorial depiction (Pepperell, 2015), even if this cubist example is an extreme case of perceptual incongruence between the pictorial fabric and what is depicted. Yet despite this dichotomy we are rarely prevented from understanding that, when looking at a picture, a certain pattern of lines or colours simultaneously stands for a quite different object.

To summarise, these cases of problem solving and art interpretation demonstrate some of the key properties of natural understanding as broadly described here, namely that it is a form of reasoning, learning or recognition that is accompanied by a consciously experienced insight, motivated by a desire to overcome anxiety and gain pleasurable reward, that entails a diverse and sometimes contradictory set of associations, some of which depend on contextual knowledge and meaning prediction, that are bound together in a simultaneous cognitive state. These features are summarised in Table 2.

**TABLE 2:** Summary of the key properties of natural understanding based on the cases of the remote associates task and the interpretation of a painting.

Key properties of natural understanding	
Insight	Aha! moment, or sudden change in how a stimulus is perceived entailing a revelation of new meaning that was previously absent
Reward	A positively valenced emotional state that intrinsically motivates effortful cognition
Learning	Adaptation by acquiring new knowledge that can be generalised to cases beyond the stimulus that produced the learning
Recognition	The ability to correctly classify a stimulus, or part of a stimulus, according to the features it presents or contains
Differentiation	The division of the perceptual stimulus into a multiple, diverse and sometimes contradictory set of meaningful elements
Integration	The unification of diverse perceptual elements into a single coherence experience, without diminishing their diversity
Context	Connecting to ideas, references and meanings that are not immediately present in the stimulus but are associated with it
Reasoning	A capacity to acquire new knowledge by logically inferring or extrapolating from existing data
Prediction	The ability to apply feedback from higher-level cognitive models to lower-level perceptual input to rapidly anticipate meaning
Consciousness	The state of being aware of the self and the environment, and in particular awareness of the stimulus and the response to it

This list does not exhaustively describe each of the properties of natural understanding, nor does it collectively provide a precise definition. And it is worth noting that some forms of understanding are arrived at by a process of logical analysis rather than sudden insight (Jung-Beeman et al., 2004; Carpenter, 2020). But, at least with respect to the cases discussed here, this list is indicative of the range of properties that natural understanding entails. Assuming we can generalise from this to other cases of natural understanding, we have identified some of the properties that an authentic implementation of machine understanding would require.

## Artificial Understanding

Having described some of the key properties of natural understanding we turn to the artificial kind as defined in the introduction. Many existing artificial intelligence systems are implemented in computational neural networks such as deeply layered convolutional neural networks that roughly approximate the function of neural cells in brain tissue. Contemporary deep neural networks evolved from early neurally inspired machine learning architectures such as the Pandemonium and the Perceptron pioneered in the 1950s (Rosenblatt, 1958; Selfridge, 1959). In these early models, continuous input data is first discretised by “feature detectors” and then passed to intervening layers of neurons that are weighted to respond to properties of the features. Based on the sum of all the weights the system reaches a decision processing about the most probable output. These models in turn inspired the later parallel distributed approaches to artificial intelligence that were developed by Rumelhart and McClelland (1986) and in many ways provided the core architecture of today’s artificial neural networks and machine learning systems.

**FIGURE 4:**
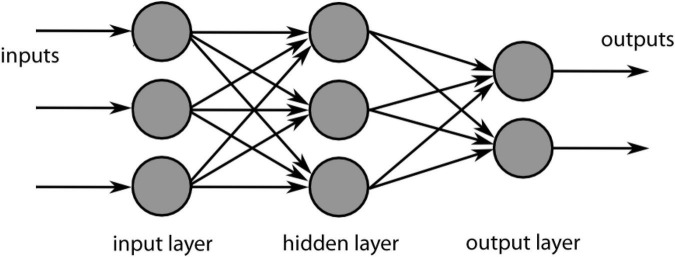
A simple feedforward neural network architecture showing an input layer that serves to discretise the target data, one hidden layer that contains nodes or “neurons” that can adjust their probabilistic weights, and an output layer where the decision of the system can be read off.

A typical artificial neural network tasked with, say, classifying objects in photographs will take an image as input, divide it into sub-sections (such as pixel colour values or clusters of pixels), pass those values to an array of nodes or neurons in one of what may be many interconnected “hidden” layers of such arrays, apply weights and biases in order to arrive at a probabilistic estimate of the likely class of the input, and pass the result to an output layer that can be read off by the user. By supplying the network with many training images, and by gradually optimising the weightings and bias using error correction techniques such as backpropagation, the network will eventually learn to classify its target objects with a degree of accuracy that depends on factors such as the size of the training dataset, the number of layers in the networks, and the amount of error correction provided. A simple feedforward example of this architecture is illustrated in Figure 4.

Since the explosion of research in artificial neural networks and deep learning techniques in the 2010s, and the accompanying exponential increase in raw computing power, a plethora of designs and methods have evolved for implementing machine learning (LeCun et al., 2015; Aggarwal, 2018). In the case of a contemporary deep learning system like BERT, the Bidirectional Encoder Representations from Transformers, several methods are combined in order to optimise performance in a range of natural language understanding tasks, with the relative performance of different variants of BERT being tested against standardised benchmarks such as SuperGLUE (Wang et al., 2019).

In these tests, passages of text are presented to humans or computers to elicit a correct answer. Different kinds of understanding are tested, including reading comprehension, choosing correctly between alternatives, or reasoning correctly based on a hypothesis. For example, in the following causal reasoning task (Roemmele et al., 2011), given the statement: “My body cast a shadow over the grass” and the question: “What’s the CAUSE for this?”, the responder must choose between alternative 1: “The sun was rising” and alternative 2: “The grass was cut,” the correct alternative being 1. In 2021, the DeBERTa variant of BERT was shown to surpass human performance against the SuperGLUE benchmark by a comfortable margin in some tests (He et al., 2021).

Image classification systems are designed to recognise, segment, or locate objects in images using convolutional neural networks that employ similar techniques to those of natural language processing systems but trained on vast databases of human annotated photographs stored on repositories such as ImageNet^5^. Competing models have been pitted against each other in contests such as the ImageNet Large Scale Visual Recognition Challenge or ILSVRC, which began in 2010 (Russakovsky et al., 2015). The ImageNet challenge uses a large dataset of annotated images from the database for training and a smaller subset for testing from which the annotations are withheld. The competing classifiers are required to perform several kinds of recognition and detection tasks on the test dataset, including predicting the classes of objects present in the image and drawing bounding boxes around objects (tasks not dissimilar to the cubist painting example discussed above). A breakthrough in image classification performance was made in 2012 with the introduction of the AlexNet architecture (Krizhevsky et al., 2017) which achieved the then unprecedented score in the ImageNet challenge of 63.3%. By 2021, systems such as Convolution and self-Attention Net (CoAtNet) were achieving accuracy scores of 90.8% (Dai et al., 2021).

Given that these natural language and image classification machines are routinely achieving 90 + % accuracy, and in some cases outperforming humans, there is a sense in which they can be rightly said to have a capacity for understanding, even though they are implemented in very different substrates from the biological tissue and processes that instantiates natural understanding. After all, show them a sentence with a missing word or a photograph containing many objects and they will reliably be able to predict the missing word or label the objects. This capacity for comprehension, reasoning, recognition, and detection implemented in digital computers is what is referred to here as artificial understanding.

The key properties of artificial understanding broadly described here are that it relies on training with large datasets through which the system learns by adjusting probabilistic weightings of the neurons, modified by error correction, resulting in statistical models that predict the most likely output for a given input, whether that is by detecting and labelling a class or reasoning from contextual data about the likely solution. To carry out this process input data is differentiated into parts and analysed to find patterns and associations between the parts which are then integrated to produce an output. These key properties of artificial understanding are summarised in Table 3.

**TABLE 3:** Summary of the key properties of artificial understanding based on the cases of natural language processing and image classification.

Key properties of artificial understanding	
Prediction	A capacity to estimate the correct output given a certain input based on probabilistic calculations
Learning	Improving performance of the system through a process of training and adaptation guided by feedback based on correctness of outputs
Differentiation	The division of the input into multiple features that can be analysed in terms of regularities and patterns
Integration	The summation of probabilistic analysis of the differentiated features to produce an output
Context	A table of statistical relationships that is extracted from the training data and used predict the most likely missing data
Recognition	Correctly identifying or labelling an object from a given input, or part of the input, by analysing its features and predicting the correct output
Reasoning	The capacity to select the correct conclusion given information that is implicit in the input but not explicitly stated

Again, this is not a comprehensive list of the key features of nor a precise definition of artificial understanding. But on the basis of the natural language processing and image classification systems discussed here we are in a position to make some instructive comparisons between the natural and artificial kinds of understanding.

## Comparing Natural and Artificial Understanding

As can be seen from Table 4, natural and artificial understanding, as described here, share several key properties, at least superficially, while some are unique to natural understanding. In this section, I compare these properties to establish how closely they are shared and what might be the significance of the differences.

**TABLE 4:** Comparison between the key properties of natural and artificial understanding based on the cases discussed above.

Comparing properties of natural and artificial understanding	
**Natural understanding**	**Artificial understanding**
**Learning**	**Learning**
**Recognition**	**Recognition**
**Differentiation**	**Differentiation**
**Integration**	**Integration**
**Context**	**Context**
**Reasoning**	**Reasoning**
**Prediction**	**Prediction**
Consciousness	
Insight	
Reward	

*Properties in bold are shared.*

### Shared Properties

*Prima facie*, both kinds of understanding share some capacity for learning, recognition, differentiation, integration, utilisation of contextual information, reasoning, and prediction. These key properties are functionally similar in humans and artificial neural networks in that for certain tasks they can produce the same outputs from the same inputs, even if the substrates they are instantiated in and the ways they are implemented are very different. In the case of natural language processing, as noted, humans and computers can achieve comparable scores when assessed against the criteria used in the SuperGLUE tests, which are based on tests designed to measure reading ability, reasoning and comprehension skills in humans (e.g., Roemmele et al., 2011). Neural network-based image classification systems also now routinely equal and sometimes out-perform humans (Buetti-Dinh et al., 2019). And neuroscientific models of predictive coding in humans have inspired new designs of neural networks with enhanced object recognition capabilities (Wen et al., 2018). All this is testament to the remarkable proficiency of artificial understanding systems in emulating these human cognitive faculties.

Yet despite the impressive levels of performance achieved with some deep learning models, and their functional similarity with human capabilities, they still differ from and fall short of human-level performance in several ways, including in terms of how robust and generalisable they are. As noted above in the case of cubist painting interpretation, humans are adept at applying what they learn in one case to novel cases (Wiesmann et al., 2009). But because deep learning systems become very finely “tuned” to the limited datasets used to train them there is a danger of “shallow” learning, where the system’s competences are limited to the training data and they are unable to adapt to new cases, as was shown recently in the domain of natural language inference (McCoy et al., 2019).

Meanwhile, image classification tasks using ImageNet-trained machine learning systems are yet to achieve human-level performance in certain tasks and are rated as being less robust and less generalisable than human agents (Shankar et al., 2020). The problems of robustness and generalisability in image classification algorithms were further highlighted by a study showing that the ability of leading models to understand the content of photographs was significantly impaired by difficult or “harder” cases, i.e., cases where the image content was more ambiguous (Recht and Roelofs, 2019).

The differences, or dissonances, between human and machine understanding (natural and artificial in the terminology used here) were explored by Zhang et al. (2019) in the context of Biederman’s theory of human image understanding (Biederman, 1985). Biederman (1985) argued that image recognition depends upon first differentiating or segmenting the image into components that are invariant with respect to viewing position or image quality and from these components the understanding of the image as a whole is constructed. Zhang et al. (2019) asked both humans and neural network (NN) image classifiers to segment a set of images into “super pixels” that contained the portions of the image most salient to recognition. They found that humans and NNs tended to segment the image in different ways. When asked to recognise objects from the segmented portions only, NNs often out-performed humans on “easy” images, suggesting that humans and NNs were using different strategies to complete the task. But NNs performed less well than humans on more difficult or ambiguous images.

Collectively, this evidence suggests that while natural and artificial kinds of understanding do share the properties listed in bold in Table 4, at least at the functional level if not at the substrate level, and have comparable levels of performance in some cases, there are significant differences in how robust and generalisable they are and in how well they are able to deal with difficult cases. Moreover, questions remain about whether machine learning systems rely on spurious correlations—that they can be “right for the wrong reasons”—and whether they genuinely have a capacity for semantic appreciation. This leaves them vulnerable to Clever Hans and Chinese Room-style criticisms, viz., that they are not, by their essential nature, authentically cognising or understanding at all.

### Unique Properties

The essential differences between natural and artificial understanding become more pronounced when we consider the key properties that are unique to natural consciousness, the most obvious being that it entails consciousness. Questions about the nature of consciousness, how it is instantiated in humans (or other creatures for that matter), and how it might be implemented in non-biological substrates are vast and deep and cannot be addressed in detail here. But it is necessary to briefly consider what the conscious property of natural understanding might be contributing to the phenomenon as a whole and why it might help to explain its essential difference from and advantages over the artificial kind. This is especially so given that two of the other key features of natural understanding as described here, namely insight and reward, are themselves aspects of conscious experience.

Consciousness can be defined as the state of awareness of self and environment, and while this begs the question of what is meant by awareness, I will take it that we are familiar with what it means in ourselves. One way to measure the difference between a system that is conscious and one that is not is that a conscious system such as a human brain displays very high levels of simultaneous differentiation and integration in its organisation and behaviour (Tononi et al., 1994). Of course, any system composed of different subsystems that are coupled together, i.e., a system of systems, will be differentiated and integrated to some degree (Nielsen et al., 2015). But in the case of the human brain this degree seems to be extremely large (Tononi et al., 1994) and far greater than in existing machine learning systems if we take the complexity of the system as a measure: it requires a convoluted neural network having seven layers to emulate the complexity a single human neuron (Beniaguev et al., 2021) and there are estimated to be around 86 billion such neurons and around the same number of non-neuronal cells in a human brain (Azevedo et al., 2009).

Recent evidence from the neuroscientific study of consciousness suggests that there is something particular about the way brain activity during conscious states is differentiated and integrated that contributes to the production of phenomenal states. The Global Neuronal Workspace Hypothesis (GNW) advocated by Baars et al. (2013) and Mashour et al. (2020) proposes a model of conscious processing in which localised, discrete and widely distributed cortical functions are integrated via reciprocally connected long-range axons. At any one time, information from one or more of these discrete functional processors can be selectively amplified and “broadcast” across the entire system, thus producing a single integrated, coherent experience for the conscious agent concerned. The Integrated Information Theory (IIT) of consciousness championed by Tononi and Koch (2015) and Tononi et al. (2016)—in some ways a competing theory to GNW—predicts that in order for a system such as a brain to be conscious it must display a high degree differentiation (by which they mean richness or diversity of information) and integration (by which they mean interdependence or interrelatedness of the information), the quantity of which is given by a value known as Φ. A fully conscious brain, for example, will contain a greater quantity of Φ than a partially conscious or unconscious brain.

Tononi and Koch point to work conducted by Casali et al. (2013) as empirical support for this hypothesis. By applying a magnetic pulse to the brains of people having varying levels of consciousness, including severely brain damaged patients showing little or no signs of conscious awareness, and then measuring the resulting patterns of activation using information-theoretical measures of complexity, the experimenters were able to reliably discriminate between levels of consciousness on the basis of how much differentiation and integration the patterns of activation displayed^6^. They found that greater levels of differentiation and integration reliably predicted higher levels of consciousness, and could predict which people were unconscious when these levels fell below a certain threshold in their brains, such as in those with severe brain damage who were in a vegetative state. It is important to note that even though the brains of people with impaired consciousness were still functioning to some extent, and therefore displaying a high degree of differentiation and integration by the standards of many physical systems, they fell short of the threshold necessary to support full consciousness.

Further evidence that fully conscious states rely on maintaining a critical balance between activity in localised and segregated networks and globally integrated networks in the brain was provided by Rizkallah et al. (2019). Using graph-theory based analysis on high-density EEG data, the team showed that levels of consciousness decreased as the level of integration between long-range functional networks also decreased while, at the same time, information processing became increasingly clustered and localised. Besides disorders of consciousness, researchers have also shown that imbalances between local segregation and global integration in brain organisation are implicated in neuropsychiatric and other clinical disorders (Fair et al., 2007; Lord et al., 2017).

One difficult question raised by this evidence is whether there is a direct causal relationship between the levels of differentiation and integration observed in the activity of the brains of conscious people and their conscious states, or whether the correlation is spurious (Pepperell, 2018). The question is too philosophically involved to be addressed in depth here. But the phenomenal character of natural understanding, as described above, which entails an awareness of both the parts of the thing understood and the relations between the parts at the same time, is but one expression what seems to be a property of all conscious states, which is that they are experienced as simultaneously differentiated and integrated, as was observed by Leibniz (1998) in the eighteenth century and by many since^7^. Although this correlation is not proof of a causal link between phenomenology and underlying neurobiology, and nor does it explain why the particular kind or degree of differentiation and integration that occurs in conscious brains is critical, it does weaken any claim that the correlation is merely spurious.

With respect to the property of insight, which is consciously experienced, there is evidence from neuropsychology that comprehension or understanding, including that which is achieved through sudden insight or Aha!, is mediated by regions of the brain that are important for integration of differentiated brain processes (St George et al., 1999; Jung-Beeman et al., 2004). The same principle has been observed in the mechanisms that bind together widely distributed brain areas as object representations become conscious (Tallon-Baudry and Bertrand, 1999). Other studies have demonstrated that the appearance of sudden moments of insight or comprehension are in fact the culmination of multiple preceding brain states and processes, suggesting that insight favours the “prepared mind” and acts to draw these largely unconscious processes together into a single conscious state (Kounios and Beeman, 2009). This evidence therefore also points to a link between the underlying mechanisms that mediate consciousness and the phenomenology of natural understanding, or insight.

With respect to the property of reward, studies on the affective states of people who experience insights consistently show that they are emotionally diverse but positively valenced, with the most reported emotional states being happiness, certainty, calm, excitation, ease and delight (Shen et al., 2016). The affective states associated with insight and problem solving have been shown to depend on activity in regions of the brain associated with positive affect and reward and on task-related motivational areas as well as being implicated in processes of learning reinforcement, memory reorganisation, semantic coherence, and fast retrieval encoding (Tik et al., 2018).

The motivating power of potential reward, even when cued subliminally, was demonstrated by researchers who used a version of the remote associate task cited above to test problem solving performance in people (Cristofori et al., 2018). Based on their results they speculated that the potential for reward activated systems of the brain that reinforce behaviour, facilitate cognition, and enhance automatic integration of differentiated processes. The fact that they did so subliminally was argued to promote overall performance because cognitive resources were not diverted from conscious processes such as attention selectivity. Further evidence shows that mood can significantly affect a person’s performance in problem solving, with people in positively valenced states of mind being able to solve problems or reach insights better than those in a less positive mood (Subramaniam et al., 2009). This finding reinforces the association between consciously experienced affect and capacity for understanding.

While is premature to draw firm conclusions from the neurobiological and psychological data relating to the key properties that are unique to natural understanding, it does seem to point toward a general trend: that the act of consciously understanding something is characterised by high degrees of simultaneous differentiation and integration—both neurobiologically and phenomenologically—and positively valenced affect that rewards problem solving and motivates learning. This comparative analysis between the shared and unique properties makes clear that although there are functional similarities between natural and artificial kinds of understanding there are also significant differences in function and in essence due, in part, to the conscious properties that natural understanding entails.

## Hypothesis

From the evidence and argument presented it is proposed that the present performance limitations of artificial understanding, and the questions about its authenticity noted in the introduction, may arise, at least in part, because it lacks the capacity for consciousness and the associated capacities for insight and reward that we find in natural understanding. This proposal can be expressed in the following hypothesis:

*The capabilities deemed desirable but deficient in artificial understanding systems, viz., robustness, generalisability, competence in hard cases and authentic appreciation of meaning, occur in natural understanding, at least in part, because the motivation to gain insight, the unification of divergent concepts that the insight entails, and the reward that comes from achieving it are consciously experienced.*

The hypothesis suggests that there may be at least two reasons why the properties unique to natural understanding contribute to its capabilities and essential nature:

1. The promise of reward, and the positive affective states entailed by achieving reward, provide the system with the intrinsic motivation (Di Domenico and Ryan, 2017) to devote the necessary cognitive resources, such as memory search, object recognition, and selective attention, to the task in hand. This in turn reinforces learning and promotes memory reorganisation which improves performance in subsequent related tasks, particularly with respect to difficult cases, while also contributing to robustness.
2. The neurobiological activity that produces high degrees of simultaneous differentiation and integration, and which is associated with the occurrence of consciousness in humans, allows the understander to assimilate many diverse cognitive states into a single overarching cognitive state without effacing the differences between its constituent states. This neurobiological activity is reflected at the phenomenological level, as described in section “Natural Understanding,” where natural understanding is characterised by the simultaneous “grasping” of diverse, and sometimes contradictory, concepts that form a meaningful conceptual whole.

Both of these reasons would require further analysis, investigation, and ideally empirical testing before we can draw any conclusions about their validity.

## Implementing Machine Understanding

The question of how to implement machine understanding is related to, but distinct from, the question of how to implement machine consciousness (Haikonen, 2003; Pepperell, 2007; Yufik, 2013; Manzotti and Chella, 2018; Hildt, 2019). It is beyond the scope of this article to consider in any detail the conceptual and technical challenges that would face someone trying to encode the properties of natural understanding, as described here, in a non-human substrate. However, if we take it that it is the *natural* form of understanding that we are seeking to implement it follows that a naturalistic approach to creating such machines may be beneficial. By ‘‘naturalistic’’ I mean an approach that seeks to model the properties and functions of the naturally occurring phenomenon as closely as possible^8^. This would be in keeping with the early models of machine learning, cited above, that were directly inspired by natural biological processes.

Even though today’s artificial neural networks are the direct descendants of these early naturalistically inspired models, they differ in important ways from the biological processes that underlie human cognition and consciousness. Consider, for example, that the adult human brain accounts for around 2% of body mass, but consumes around 20% of the body’s energy budget when at rest, or some 20 W (Sokoloff, 1992; Laughlin, 2001). Yet while this might suggest that the brain is extremely energy hungry it is in fact extraordinarily efficient when compared to current day computers, especially those carrying out machine learning tasks (García-Martín et al., 2019). Training just one learning model just once can consume over 600,000 kWh (Strubell et al., 2019) while the amount of power (in terms of ATP availability) used by the cerebral cortex to carry actual computation has been estimated at around 0.1 W (Levy and Calvert, 2021).

Consider also that the organisation and exploitation of energy resources by the brain may be playing a far more significant role in the production of consciousness than is often assumed (Shulman, 2013). It can be argued that neuroscientific models of brain activity based primarily on digital information processing paradigms, which tend to predominate in the current literature, have underplayed the causal role of energy in the production of phenomenological states (Pepperell, 2018). For example, the groundbreaking work on measuring consciousness based on levels of differentiation and integration by Casali et al. (2013) noted above is commonly interpreted in information theoretical terms, where greater “information processing” relates to greater consciousness. Yet the same results could be equally well interpreted in energetic terms on the basis that greater levels of differentiation and integration of the metabolic processes in the brain are causally related to the greater levels of consciousness observed.

Recent attempts have been made to dramatically improve the energy efficiency of machine learning systems using neuromorphic hardware (Stöckl and Maass, 2021) and given the growing awareness of the environmental impact of machine learning computing this is likely to become a topic of more intense research (Dhar, 2020). Alongside this there is growing interest in better understanding the causal role that energy and work plays in mental functions like understanding (Yufik et al., 2017) and in thermodynamically inspired models of computing which attempt to harness the natural computational power of complex, self-organising, non-equilibrium systems (Hylton, 2020). At the same time arguments continue about whether the physical substrate in which any form of machine understanding or consciousness is implemented might have a critical bearing on its functionality and efficiency (Koene, 2012). Such arguments become especially relevant in the context of a naturalistic approach where, for example, the foundational role of energy acquisition and dissipation in artificial intelligence is highlighted (Thagard, 2022). These developments suggest that considerations about the role that energy is playing in the natural system of the brain will increasingly inform future development of machine understanding and machine consciousness.

There is also an active line of research into designing systems capable of human-like faculties of perception, cognition and consciousness that is directly inspired by current neuroscientific theories of brain function (Marblestone et al., 2016). Prominent among these are models based on the Global Neuronal Workspace (GNW) theory cited above (Haqiqatkhah, 2019; Mallakin, 2019; Safron, 2020; VanRullen and Kanai, 2021). According to this theory, the brain contains many processes that are highly differentiated, localised, widely distributed and yet unconscious. Under certain conditions, these localised processes are broadcast across the entire brain network to form an integrated cognitive state which advocates of the theory argue is experienced consciously. Relating this theory to the example discussed in section “Natural Understanding,” we could imagine the diverse perceptions, concepts, and associations generated by the cubist painting being instantiated in such distinct cortical processes across the brain. At the same time, the richly interconnected global workspace area containing long-distance axons is able to select one or more local processes to be broadcast to the entire system, thus allowing for widespread and simultaneous integration of the diverse processes, just as we experience when we have gained an understanding of the painting’s meaning. Researchers such as VanRullen and Kanai (2021) have proposed methods for implementing the GNW in artificial neural networks with a view to improving the performance of current machine learning systems and potentially endowing them with a capacity for consciousness. If validated such brain-inspired machines would, in principle, satisfy the requirements for a mechanical implementation of natural understanding as defined here.

However, there are also reasons to be cautious about our ability to emulate natural understanding given the limitations of current computer architectures and therefore our ability to replicate natural processes in machines. A key property of the brain activity associated with consciousness is the presence of highly recursive neural processing in which activity is fed forward and backward throughout the brain, creating dynamic loops that bind local processes into larger global networks. GNW is one of several theories of brain function that foreground the importance of recursive, reentrant or recurrent processing (Edelman and Gally, 2013; Lamme, 2020) and diminution of such feedback activity has been shown to be one of the hallmarks of loss of consciousness during anaesthesia (Lee et al., 2009; Hudetz and Mashour, 2016). According to GNW, recurrent processing is one mechanism through which the simultaneity of conscious experience, in which multiple and diverse contents are bound into a single state of mind, is generated (Mashour et al., 2020). Given the highly complex physiological organisation of the brain, noted above, with its billions of interacting cells densely arranged in a three-dimensional lattice, it is not hard to appreciate how intricate multiscalar patterns of recurrent processing occur.

It is much harder to imagine how similar levels of recurrent processing could be implemented, or even simulated, in today’s digital computer architectures. The physical design and operation of current computer hardware, which is generally controlled by a central processing unit that executes lines of computer code sequentially at a fixed clock rate, means that it is incapable of producing the highly non-linear and globally interconnected behaviour we observe among biological neurons. Moreover, the primarily linear nature of programme execution in current computers (notwithstanding parallel processing architectures) mitigates against the simultaneity of processing that seems to mark natural understanding and conscious processing. Of course, software-implemented feedback mechanisms are often integral to machine learning algorithms (Herzog et al., 2020) and neural feedback can be simulated in software (Caswell et al., 2016). Moreover, recent research into how recurrent processing in mammalian brains aids object recognition has also shown that it improves performance when simulated in neural nets (Kar et al., 2019). But generating the degree of recurrent and simultaneous processing necessary to support the synchronised integration of highly numerous and diverse modules, in the way that seems to mark understanding and consciousness in humans, may be far beyond the capability of current digital computer architectures given the requirement for complexity noted above.

This brief survey suggests that while natural biological processes continue to be a source of guidance and inspiration for those seeking to implement humans cognitive faculties such as consciousness in non-human substrates significant challenges and problems remain to be overcome.

## Conclusion

This article addressed the question of whether consciousness is required for machine understanding. I have shown that although we lack a precise operational definition of understanding we can draw a useful distinction between the natural, artificial and machine kinds. By analysing concrete examples of natural understanding I have described some of its key properties and contrasted these with some of the key properties of artificial understanding. Although much more could be said about these properties and the contrasts between them, it is evident from the analysis presented here that the conscious properties of natural understanding mark a profound difference in both function and essence from artificial understanding, even though both share some functional similarities.

On the basis of this analysis, I have proposed a hypothesis that may help to explain the advantages that natural understanding has over the artificial kind, specifically in terms of its capacity for robustness and generalisability, its ability to deal with difficult cases, and in the authenticity of its cognitive and semantic processing. The practical challenges of implementing machine understanding have been briefly considered, and are clearly considerable. I suggest that a naturalistic approach to addressing this challenge may be beneficial, which means modelling the biological processes and structures that mediate understanding in humans and implementing these as efficiently as possible in a non-human mechanical substrate. However, pursuing this approach may require us to move beyond today’s computational architectures.

There are several limitations of the present study. To mention three: first, as stated at the outset, the phenomenon of natural understanding is highly complex and multifaceted, and we lack any precise definition of what understanding is. Worse, different people in different disciplines can take it to mean different things. As such, it is unlikely that any single analysis will be able to capture all its many psychological and neurobiological properties, define them all in detail, and explain how they all interact in a way that all agree upon. The pragmatic approach taken here has been to describe these properties in broad terms rather than define them precisely to provide a useful working account of the phenomenon so that it can be compared to other implementations of understanding in certain cases. But any future work in this area will inevitably require more precise and generally agreed definitions.

Second, the relationship between consciousness and understanding as discussed here is complicated by the fact that many of the cognitive processes that enable natural understanding occur subliminally, as noted above. Future investigations may need to take greater account of the role of unconscious processing in the brain, and how this might inform the design of machine understanding systems. This raises further questions about the extent to which we need to replicate natural brain processes and functions to successfully implement human-like capabilities in non-human substrates or whether designing machines that achieve more or less the same results, even if by very different means, will be sufficient “for all practical purposes” (Anderson, 2017).

Third, the problem of machine understanding is one that, to date and to a large extent, has been addressed within the discipline of computer science. The analysis presented in this article is highly interdisciplinary, drawing on knowledge from art history, psychology, neuroscience, computer science, consciousness studies and other fields. There is always a danger in such highly interdisciplinary studies of oversimplifying its constituent knowledge. However, the problem of machine understanding may be one that is so broad and so deep that we have no option but to take such a highly interdisciplinary approach. In which case we will need to establish protocols of cooperation among widely dispersed areas of research.

## Data Availability Statement

The original contributions presented in the study are included in the article/supplementary material, further inquiries can be directed to the corresponding author.

## Author Contributions

The author confirms being the sole contributor of this work and has approved it for publication.

## Conflict of Interest

The author declares that the research was conducted in the absence of any commercial or financial relationships that could be construed as a potential conflict of interest.

## Publisher’s Note

All claims expressed in this article are solely those of the authors and do not necessarily represent those of their affiliated organizations, or those of the publisher, the editors and the reviewers. Any product that may be evaluated in this article, or claim that may be made by its manufacturer, is not guaranteed or endorsed by the publisher.

## Footnotes

1 A mechanical substrate is taken here to be a system composed of electrical and mechanical components that is designed to enable the processing of understanding, such as a computer or robotic system, that can receive data as input and produce a readable output.

2 The answer in each case is 1. BLUE, 2. POST, and 3. BIRTH. In the paper from which these examples are taken 10% of the participants tested were able to find the correct answer to 1 in less than 2 s, while only 1% were in the case of 2 and none were in the case of 3 (Bowden and Jung-Beeman, 2003).

3 There is also evidence that learning to understand cubist paintings by recognising the objects in them increases people’s aesthetic experience of the paintings (Muth et al., 2013).

4 It is not clear from this illustration whether the collection of ideas and associations contained in the viewer’s thought bubble are being experienced simultaneously or sequentially. Personal experience of studying artworks in this way suggests that it is probably a mixture of both.

5
http://www.image-net.org

6 The measure of complexity in this case was the compressibility (using the Lempel-Ziv algorithm) of the data generated by imaging the perturbation in the brains due to the magnetic pulse (Ziv and Lempel, 1977).

7 Leibniz (1998) noted on several occasions that perception is “the expression of a multitude in a unity.” More recently, Guilio Tononi, one of the prime movers behind IIT, stated: “consciousness corresponds to the capacity of a system to integrate information. This claim is motivated by two key phenomenological properties of consciousness: differentiation – the availability of a very large number of conscious experiences; and integration – the unity of each such experience” (Tononi, 2004).

8 For an example of a naturalistic approach applied to the problem of computationally modelling human visual space see Burleigh et al. (2018).
